# Biochemical Characterization and Validation of a Catalytic Site of a Highly Thermostable Ts2631 Endolysin from the *Thermus scotoductus* Phage vB_Tsc2631

**DOI:** 10.1371/journal.pone.0137374

**Published:** 2015-09-16

**Authors:** Magdalena Plotka, Anna-Karina Kaczorowska, Agnieszka Morzywolek, Joanna Makowska, Lukasz P. Kozlowski, Audur Thorisdottir, Sigurlaug Skírnisdottir, Sigridur Hjörleifsdottir, Olafur H. Fridjonsson, Gudmundur O. Hreggvidsson, Jakob K. Kristjansson, Slawomir Dabrowski, Janusz M. Bujnicki, Tadeusz Kaczorowski

**Affiliations:** 1 Department of Microbiology, University of Gdansk, Gdansk, Poland; 2 Collection of Plasmids and Microorganisms, University of Gdansk, Gdansk, Poland; 3 Faculty of Chemistry, University of Gdansk, Gdansk, Poland; 4 Laboratory of Bioinformatics and Protein Engineering, International Institute of Molecular and Cell Biology, Warsaw, Poland; 5 VESO Vikan, Namsos, Norway; 6 Matis, Reykjavik, Iceland; 7 ORF genetics, Kopavogur, Iceland; 8 Faculty of Life and Environmental Sciences, University of Iceland, Reykjavik, Iceland; 9 Prokazyme ehf, Reykjavik, Iceland; 10 A&A Biotechnology, Gdynia, Poland; 11 Laboratory of Bioinformatics, Institute of Molecular Biology and Biotechnology, Faculty of Biology, Adam Mickiewicz University, Poznan, Poland; ContraFect Corporation, UNITED STATES

## Abstract

Phage vB_Tsc2631 infects the extremophilic bacterium *Thermus scotoductus* MAT2631 and uses the Ts2631 endolysin for the release of its progeny. The Ts2631 endolysin is the first endolysin from thermophilic bacteriophage with an experimentally validated catalytic site. *In silico* analysis and computational modelling of the Ts2631 endolysin structure revealed a conserved Zn^2+^ binding site (His^30^, Tyr^58^, His^131^ and Cys^139^) similar to Zn^2+^ binding site of eukaryotic peptidoglycan recognition proteins (PGRPs). We have shown that the Ts2631 endolysin lytic activity is dependent on divalent metal ions (Zn^2+^ and Ca^2+^). The Ts2631 endolysin substitution variants H30N, Y58F, H131N and C139S dramatically lost their antimicrobial activity, providing evidence for the role of the aforementioned residues in the lytic activity of the enzyme. The enzyme has proven to be not only thermoresistant, retaining 64.8% of its initial activity after 2 h at 95°C, but also highly thermodynamically stable (T_m_ = 99.82°C, ΔH_cal_ = 4.58 × 10^4^ cal mol^-1^). Substitutions of histidine residues (H30N and H131N) and a cysteine residue (C139S) resulted in variants aggregating at temperatures ≥75°C, indicating a significant role of these residues in enzyme thermostability. The substrate spectrum of the Ts2631 endolysin included extremophiles of the genus *Thermus* but also Gram-negative mesophiles, such as *Escherichia coli*, *Salmonella panama*, *Pseudomonas fluorescens* and *Serratia marcescens*. The broad substrate spectrum and high thermostability of this endolysin makes it a good candidate for use as an antimicrobial agent to combat Gram-negative pathogens.

## Introduction

It has been estimated that approximately 10^30^−10^32^ phages, or viruses that infect bacteria (also called 'bacteriophages'), exist in the biosphere and cause approximately 10^23^ infections per minute [1–3]. At the last stage of infection, phages, like other viruses, release their progeny from the host cells. This is achieved through either extrusion of new viral particles in a secretory process or by disruption of the bacterial cell wall leading to host cell lysis. The first mechanism is used by filamentous phages that are continuously exported from bacterial cells without killing them, whereas nonfilamentous bacteriophages induce lysis of the host cell, mostly with the use of phage-encoded enzymes called endolysins [4]. Endolysins target specific bonds within bacterial peptidoglycan, which is the major structural component of the bacterial cell wall. Peptidoglycan maintains the shape of the cell and is essential for bacteria to resist the high intracellular turgor pressure [5, 6]. Peptidoglycan consists of long glycan strands cross-linked by short peptides [7]. The glycan chain is a polymer of alternating N-acetylmuramic acid (MurNAc) and N-acetylglucosamine (GlcNAc) residues linked together by β-1,4- glycosidic bonds. The glycan component is covalently linked to the short stem peptide through an amide bond between MurNAc and an L-alanine. The basic stem peptide sequence is L-Ala-D-Glu-DAP-D-Ala-(-D-Ala). The *meso*-diaminopimelic acid (DAP) residue is embedded into the peptidoglycan of most Gram-negative bacteria (known as the so called DAP-type peptidoglycan) but is substituted with L-lysine (L-Lys) in most Gram-positive species (Lys-type peptidoglycan) and with L-ornithine (L-Orn) in *Thermus thermophilus*, Spirochetes and *Bifidobacterium globosum* [7].

The diversity of the complex peptidoglycan architecture in Gram-negative or Gram-positive bacteria is reflected by the pronounced functional and structural variety of phage-encoded endolysins [8]. Endolysins from phages infecting Gram-negative species are usually small globular proteins (15–20 kDa) and possess only an enzymatically active domain (EAD) [6]. Endolysins specific for Gram-positive bacteria are larger, modular proteins with two separate domains: an EAD and a cell wall binding domain (CBD) [9]. The EAD confers the lytic activity and is responsible for the cleavage of the peptidoglycan polymer [6]. Depending on the type of bond targeted in the peptidoglycan structure, endolysins may be divided into five classes: muramidases (also known as lysozymes), glycosaminidases, endopeptidases, amidases, and lytic transglycosylases [4, 5].

Amidases are among the most abundant and the earliest identified endolysins. They hydrolyse the amide bond between the sugar (MurNAc) and the peptide (L-Ala) moieties [6]. Amidases appear to be the most universal endolysins as the amide bond is highly conserved in peptidoglycan structures [10]. In bacteriophages from the genus *Thermus*, two amidases has been identified to date: the putative phage P23-77 endolysin form *T*. *thermophilus* [11] and a highly thermoresistant phage Ph2119 endolysin from *T*. *scotoductus* MAT2119 [12]. The latter was predicted to possess an *Amidase_2* domain characteristic of T7 and T3 lysozymes, which is homologous to PGRP domain of eukaryotic peptidoglycan recognition proteins (PGRPs). The eukaryotic PGRPs play a role in host defence as innate immunity pattern recognition and effector molecules. Both the *Amidase_2* domain of phage endolysins and the PGRP domain of eukaryotic recognition proteins have similar structures that consist of three peripheral α-helices and several central β-strands. In the front of the molecule there is a cleft that forms a peptidoglycan-binding groove. In the cleft, PGRPs exhibiting amidase activity possess a highly conserved Zn^2+^ binding catalytic site that consists of two histidines, one cysteine and one tyrosine [13–15]. The aim of our study was to characterize a novel, globular endolysin from the *Thermus scotoductus* phage vB_Tsc2631 comprising an EAD domain only. This domain displayed sequence similarity to the thermophilic Ph2119 endolysin and structural similarity to T7 and T3 lysozymes and to eukaryotic PGRP domain. Based on the comparative analysis and molecular modelling, we have selected residues putatively responsible for the lytic activity of the Ts2631 endolysin. In the present study, we experimentally investigated the role of the selected residues in the catalytic activity of the enzyme. To our knowledge, the Ts2631 endolysin is the first thermophilic endolysin with an experimentally investigated active site. Moreover, in the era of growing antibiotic resistance there is a need to develop new alternative ways to combat pathogenic bacteria. Biochemical, substrate specificity and stability tests of the Ts2631 endolysin were performed and revealed a potential role for the enzyme in combating Gram-negative pathogens.

## Materials and Methods

### Bacterial strains, media and plasmids

The *Escherichia coli* strains DH5α and BL21(DE3) (Invitrogen) were used for molecular cloning and protein overproduction, respectively. The bacteria were cultivated in Luria broth (LB) or Luria agar (LA) medium [16]. When necessary, the media were supplemented with ampicillin (Ap) 100 μg ml^-1^ or chloramphenicol (Cm) 30 μg ml^-1^. *Thermus scotoductus* MAT2631 was isolated on medium 166 [17]. The bacteria used as substrates for the Ts2631 endolysin were purchased either from Deutsche Sammlung von Mikroorganismen und Zellkulturen GmbH, Germany (*T*. *thermophilus* HB8, DSM 579, *Pseudomonas fluorescens*, DSM 50090, *Clostridium sporogenes* DSM 767) or the American Type Culture Collection (*Bacillus cereus*, ATCC 13061; *Bacillus subtilis*, ATCC 6633; *Deinococcus radiodurans*, ATCC 13939; *Staphylococcus aureus*, ATCC 25923; *Staphylococcus epidermidis*, ATCC 12228). Other bacteria used were taken either from the Collection of Plasmids and Microorganisms, University of Gdansk, Poland (*Escherichia coli* MG1655, ATCC 47076; *Salmonella* serovar *Panama*, KPD101-B; *Serratia marcescens*, KPD102-BA; *Enterococcus faecalis*, KPD100-BA; *Staphylococcus intermedius*, KPD105-BA; *Micrococcus luteus*, KPD104-BA; *Lactococcus lactis*, KPD103-BA) or the MATIS collection of microorganisms (*Thermus scotoductus*, MAT2119; *Thermus scotoductus* MAT2631, *Thermus flavus*, MAT1087). *Clostridium sporogenes* DSM 767 was cultivated anaerobically in Thioglycollate broth with Resazurin (bioMerieux) at 37°C without shaking. The following vectors were used in this work: pUC18/19 (Ap^R^) (Thermo Scientific) and pET15b (Ap^R^) (Novagen). The chloramphenicol-resistant plasmid pRARE, a derivative of pACYC184, was used to supply tRNAs for the rare codons: AGG, AGA, GGA, AUA, CUA and CCC (Novagen). The plasmid pLT1 overexpressing the Ts2631 endolysin gene, as well as plasmids used for overproduction of the Ts2631 endolysin variants, were constructed in the present study and were deposited in the Collection of Plasmids and Microorganisms, University of Gdansk, Poland.

### Isolation and sequencing of phage Tsc2631

The water sample from which phage Tsc2631 was isolated was incubated with different thermophilic strains, including *Thermus scotoductus* MAT2631. An overnight culture (1 ml) was mixed with 4 ml of the water sample passed through a 0.22 μm filter, and the suspension was incubated at 65°C for 30 min. Then, 5 ml of soft agar with 10 mM MgCl_2_ was added and poured onto medium 166 solidified with agar [17]. The plates were incubated at 65°C overnight, and the resulting plaques were aseptically transferred to Eppendorf tubes with 100 μl 10 mM MgCl_2_ and were stored at 4°C. The plaque solution was diluted 10^3^ to 10^4^ with 10 mM MgCl_2_, and 100 μl of the diluted phage solution was added to 900 μl of an overnight culture of *T*. *scotoductus* MAT2631 and was kept at 65°C for 30 min. Next, 3 ml soft agar was added, and the mixture was poured onto plates with medium 166 agar. After overnight growth, the plaques were picked and the whole procedure was repeated four times to purify the phage particles. The phage isolated using this procedure was designated vB_Tsc2631 (for short Tsc2631), according to the newly proposed nomenclature [18]. Phage amplification was achieved by using a 10^3^ to 10^4^ phage dilution. The soft agar was scraped from 20 plates and was placed into 100 ml of 10 mM MgCl_2_. The mixture was incubated at room temperature for 2 h with shaking (600 rpm) and was then centrifuged at 11,000 × g for 20 min. The supernatant was filtered through a 0.22 μm filter and the titre was analysed. Phage DNA was isolated from the supernatant with a titre > 10^9^ pfu ml^-1^ using a lambda midi kit (Qiagen #12543) according to the manufacturer's instructions. The genome of the phage Tsc2631 was fragmented, and the resulting DNA fragments (600–800 bp) were subcloned into pUC18/19 vectors followed by automated Sanger sequencing of the inserts. The sequence reads were assembled into contigs using Sequencher (Gene Codes Corporation, Ann Arbor, MI USA), which is available through the website http://www.genecodes.com.

### Computational analysis and molecular modelling

The nucleotide and protein sequences were analysed using the BLAST and DELTA-BLAST programs [19] available on the National Center for Biotechnology Information web page (http://www.ncbi.nlm.nih.gov). The protein sequences were aligned using the T-Coffee computer program [20] and the CLUSTAL W program [21] accessible through the European Bioinformatics Institute server (http://www.ebi.ac.uk). The isoelectric point (pI) and molecular weight of the Ts2631 endolysin was predicted using the Isoelectric Point Calculator (http://isoelectric.ovh.org). The GeneSilico Fold Recognition metaserver [22] was used to predict the protein secondary structure and to find templates for protein structure prediction by comparative modelling. As zinc ions are crucial for the activity of endolysins from the family of enzymes related to the Ts2631 endolysin [23], we restricted our choice to templates with Zn^2+^ (26 out of 75 possible templates). Based on the combined criteria of high sequence identity, high scores from the GeneSilico metaserver and high resolution of the experimentally determined structures, we selected the crystal structure of the *Drosophila melanogaster* peptidoglycan recognition protein LB (PDB ID 1OHT [24]) as the potential best template for Ts2631 endolysin molecular modelling. Comparative modelling of the Ts2631 endolysin structural core (including the active site) was performed using the Swiss-PDBViewer [25] followed by addition of the termini (missing from the template structure) using MODELLER [26].

### Cloning of the gene encoding the Ts2631 endolysin

The *ts2631* gene was cloned from genomic DNA of phage Tsc2631 by PCR amplification, digestion with restriction enzymes and ligation into vector pET15b, which carries an N-terminal His-tag sequence. The PCR primers were designed using the Primer3plus programme (http://primer3plus.com/) based on the DNA sequence of the gene. NdeI and BamHI restriction sites were added to the forward and reverse primers, respectively (S1 Table). Thermostable Platinum *Pfx* polymerase was used for PCR amplification (Invitrogen). The resulting PCR product was digested with NdeI and BamHI and was ligated into the expression vector pET15b. The ligation product, named pLT1, was introduced into *E*. *coli* BL21(DE3)[pRARE] by chemical transformation [27], and the DNA sequence of the recombinant clone (pLT1) was verified by automated DNA sequencing.

### Site-directed mutagenesis

Five residues (His^30^, Tyr^58^, His^131^, Thr^137^ and Cys^139^) of Ts2631 endolysin were substituted with Asn, Phe, Asn, Lys and Ser, respectively, and the resulting protein variants were designated as H30N, Y58F, H131N, T137K and C139S. Site-directed mutagenesis was performed according to the QuikChange II Site-Directed Mutagenesis Kit manual (Agilent Technologies). The primers used in this study are listed in S1 Table. The recombinant plasmid pLT1 was used as the PCR template, and thermostable PrimeSTAR GXL DNA polymerase (Takara Bio) was used for PCR amplification. The PCR products were treated with DpnI enzyme (1U) for 1 h at 37°C to digest the *in vivo*-methylated parental template DNA and were then introduced into *E*. *coli* DH5α cells. The recombinant plasmids carrying the mutated *ts2631* gene isolated from *E*. *coli* DH5α cells were verified for the presence of the correct mutation by DNA sequencing and were introduced into *E*. *coli* BL21(DE3)[pRARE] by chemical transformation.

### Overproduction and purification of the Ts2631 endolysin

To overproduce the Ts2631 endolysin, *E*. *coli* BL21(DE3) cells harbouring pRARE and pLT1 were cultivated at 37°C in LB medium (1 l) supplemented with Ap and Cm. When the turbidity at 600 nm reached 0.5, isopropyl-β-D-thiogalactopyranoside (IPTG) was added to the culture to a final concentration of 1 mM, and the cells were further cultured at 37°C for 4 h. The cells were harvested by centrifugation (10,000 × g; 20 min) and were stored at -80°C until further use. The thawed cell paste (4 g) was suspended in 30 ml of NPI buffer (50 mM NaH_2_PO_4_, pH 8.0, 300 mM NaCl, 10 mM imidazole, 0.1% Triton X-100, 10% [vol/vol] glycerol, 2 mM 2-mercaptoethanol [ME], 1 mM phenylmethylsulfonyl fluoride [PMSF]), and was disrupted by sonication (30 bursts of 10 s at an amplitude of 12 μm). The cellular debris was removed by centrifugation (10,000 × g; 20 min), and the clarified lysate was treated for 15 min at 75°C followed by centrifugation (10,000 × g; 20 min). The clear lysate was gently agitated with 2 ml of TALON cobalt metal affinity resin, and the purification procedure was performed according to the protocol for batch/gravity-flow column purification (Clontech, Mountain View, CA, USA). Elution of bound protein was performed with five column volumes of NPI buffer containing 150 mM imidazole. The pooled fractions containing Ts2631 endolysin were dialysed against buffer D (25 mM potassium phosphate buffer pH 8.0, 50 mM KCl, 0.1% Triton X-100, 10 mM ME and 50% glycerol) and were stored at -80°C. The Ts2631 substitution mutants were purified as described above without the heat treatment step. The purity of the recombinant endolysin was monitored by SDS-PAGE (12.5%) and size exclusion chromatography on a Superose 12 10/300 GL column using the Agilent 1100 HPLC System (GE Healthcare). Protein concentrations were determined with the Bradford reagent (SIGMA Aldrich) using BSA as a standard [28].

### Turbidity reduction and zymogram assays

The turbidity reduction assay was performed at 60°C in a standard 96-well titration plate (Corning). The substrate bacteria, *T*. *thermophilus* HB8, were prepared as previously described [29]. Briefly, late-exponential phase bacterial cells were spun down and subsequently incubated for 45 min with chloroform-saturated 50 mM Tris-HCl, pH 7.7 at room temperature. The cells were then washed to remove residual chloroform and were stored at -80°C. Before the experiment, bacterial substrate cell pellet was suspended in a reaction buffer consisting of 10 mM potassium phosphate buffer pH 8.0, 100 mM NaCl, and the OD_600_ was adjusted to 1.0 at a 1 cm path length. To measure the endolysin activity, 10 μl of Ts2631 endolysin at the concentration range of 0.25 to 25 μg/ml (0.125 to 5 μg of protein per reaction) or 10 μl of the reaction buffer (negative control) was mixed with 190 μl of the cell substrate suspension, and the change in OD_600_ was recorded over time. Addition of the endolysin resulted in a decrease in the optical density of the cell suspension, which was measured spectrophotometrically in an EnSpire multimode plate reader (PerkinElmer). The experiments were repeated in triplicate. Negative controls with reaction buffer only were subtracted from the sample measurement. The lytic activity was calculated after the indicated time points as follows: {ΔOD_600_ sample (endolysin added)– ΔOD_600_ (buffer only)}/ initial OD_600_. Zymogram analysis was performed by using 10% SDS-polyacrylamide gels containing 0.2% (wt/vol) *T*. *thermophilus* HB8 substrate in the lower gel. After electrophoresis, the gel was gently shaken at 37°C for 16 h in 50 ml of 25 mM phosphate buffer (pH 8.0) solution containing 1% Triton X-100 to allow Ts2631 endolysin renaturation. A clear band resulting from lytic activity was visualized after staining with 1% (wt/vol) methylene blue in 0.01% (wt/vol) KOH and subsequent destining with distilled water.

### Biochemical characterization of Ts2631 endolysin

To evaluate the role of the divalent metal cations in catalytic function of the endolysin, chloroform-treated *T*. *thermophilus* HB8 cells served as a substrate. Ts2631 (5 μg) was incubated with 5 mM EDTA for 30 min at 60°C to chelate and remove any residual metal ions. Subsequently, the endolysin was dialyzed overnight against 10 mM potassium phosphate buffer (pH 8.0). Lysis of *T*. *thermophilus* HB8 cells by EDTA-treated endolysin was monitored without (control) and with the addition of divalent metal ions (CoCl_2_, NiCl_2_, ZnCl_2_, FeCl_2_, Mn(CH_3_COO)_2_, MgCl_2_, CaCl_2_) at final concentrations of 0.1 and 1 mM. The lytic activities were compared to those without EDTA treatment and cation substitution. The enzyme activity optimum was determined over a pH range of 3.0 to 12.0 in 10 mM buffers supplemented with 100 mM NaCl: glycine-HCl pH 3.0; sodium acetate pH 5.0; potassium phosphate buffer pH 6.5, 7.0 and 8.0; glycine-NaOH pH 9.0 and 10.0; Na_2_HPO_4_-NaOH pH 11.0 and 12.0. The influence of NaCl on lytic activity of the Ts2631 endolysin was tested with the addition of different concentrations of NaCl (0–1000 mM). Different temperatures (10–99°C) were applied to test the effect of temperature on Ts2631 endolysin activity. The thermal stability experiments were conducted as described previously [12]. The thermal stability of the Ts2631 endolysin was compared to the stability of hen egg white lysozyme (HEWL) (5 μg; Sigma-Aldrich).

### Differential Scanning Calorimetry (DSC)

Calorimetric measurements were obtained with a VP-DSC microcalorimeter (MicroCal) at a scanning rate 90°C/1 h. The scans were obtained at a 25 μM protein concentration. The cell volume was 0.5 ml. All of the scans were run at pH 6.0 in 20 mM MES buffer at a temperature range of 5°C to 110°C. The reversibility of the transition was checked by cooling and reheating the same sample. These measurements were recorded three times. The results from the DSC measurements were analysed using Origin 7.0 software from MicroCal, employing routines included with the instrument [30, 31]. The quantity measured by DSC is the difference between the heat capacity of the MES buffer-protein solution and that of pure MES buffer. To perform a DSC measurement of protein unfolding, the reference cell was filled with buffer, and the sample cell was filled with the protein solutions. When a protein unfolds during DSC measurements the absorption of heat that occurs causes a temperature difference (ΔT) between the cells. The reference (MES buffer) and sample solutions (Ts2631 endolysin) were equilibrated with dissolved air before being introduced into the cells. Five minutes of vacuum treatment was required to degas all of the samples. The first step in calibration was to carry out the buffer/buffer scans with the run parameters exactly the same as for the comparative scans when the protein is present in the sample cell. A pre-scan thermostat period of 15 min was used as recommended. The reversibility of the transition in pure buffer was checked by cooling and reheating the reference sample.

### Nucleotide sequence and protein structure accession number

The nucleotide sequence of Ts2631 endolysin was deposited to GenBank under the accession number KJ561354. The coordinates of the molecular model are available at ftp://genesilico.pl/iamb/models/Ts2631/

## Results and Discussion

### Structural analysis of the Ts2631 endolysin

**Fig 1:**
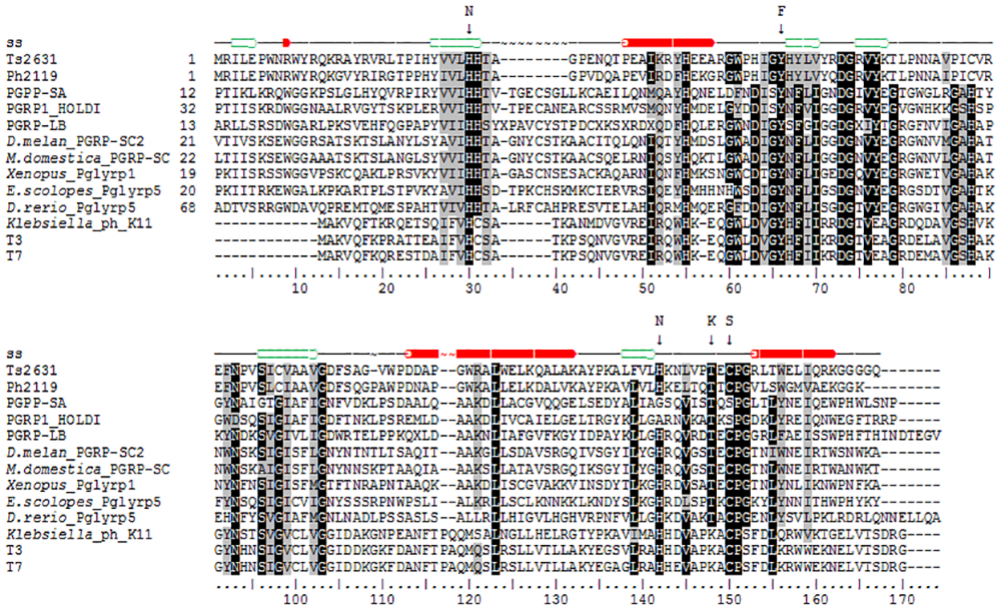
Multiple alignment of amino acid sequence of phage endolysin Ts2631, eukaryotic peptidoglycan recognition proteins (PGRPs) and endolysins of *E*. *coli* and *Klebsiella* phages T7, T3 and K11, respectively. The alignment was performed using CLUSTAL W. Grey shading reflects the amino acid conservation at 70% consensus, black boxes represent 100% aa sequence identity. All of the above proteins, regardless of their origin and natural function, display amidase activity with the exception of PGPR-SA and HOLDI that within a Zn^2+^ binding motif have serine residue instead of cysteine. Amino acid substitutions are indicated by arrows with a corresponding amino acid residue introduced via site-directed mutagenesis as described in experimental procedures. The numbers indicate the amino acids positions relative to the N-terminus. The secondary structure (ss) of Ts2631 endolysin predicted by Genesilico Fold Recognition metaserver is presented at the top line (the red cylinders represent α-helices; the green arrows represent β-strands). The accession numbers for the protein sequences of the Ts2631 endolysin, Ph2119 endolysin, PGPP-SA, PGRP1_HOLDI, PGRP-LB, *D*. *melanogaster* PGRP-SC2, *Musca domestica* PGRP-SC, *Xenopus* Pglyrp1, *Euprymna scolopes* Pglyrp5, *Danio rerio* Pglyrp5, *Klebsiella* phage K11 lysozyme, T3 and T7 lysozyme deposited in the GenBank database are KJ561354, KF408298, PDB 1SXR, Q765P4.1, PDB 1OHT, CAD89180.1, AGE46001.1, NP_001025626.1, AIR71819.1, ABE01405.1, YP_002003804.1, P_523313.1, AAB32819.1, respectively.

The bacteriophage Tsc2631 was isolated from a hot spring water sample in Hveragerdi in the south of Iceland. The selected phage host originated from a hot spring in Hrafntinnusker in the highlands of Iceland. The Tsc2631 genome was sequenced and was analysed with a suite of BLAST and DELTA-BLAST computer programs to search for open reading frames (ORF) coding for proteins with potential lytic activity. As a result, we found that the phage genome carried a 468 bp ORF coding for a protein of 156 amino acids, having an isoelectric point at pH 9.64 and a calculated molecular mass of 18,066 Da. The selected ORF was 76% identical to the extremophilic *Thermus scotoductus* phage Ph2119 endolysin. This is the first case of thermophilic phage endolysins with such a high amino acid sequence identity (74%) [12, 32]. The Ts2631 endolysin does not show any similarity to other known thermophilic enzymes with lytic activity (data not shown). The enzyme shared only approximately 25% identity when compared to enterobacterial phage lysozymes belonging to the T7 family and only 23% identity to the peptidoglycan recognition protein PGRP-SA from *D*. *melanogaster* (Fig 1). According to Pfam classification, the Ts2631 endolysin belongs to a family of N-acetylmuramoyl-L-alanine amidases (EC 3.5.1.28) and possesses a PF01510 domain (*Amidase_2*). This domain is characterized by a Zn^2+^- coordination site, which in case of T7 lysozyme, a representative member of a phage endolysin family, consists of two histidines (His^17^, His^122^), one cysteine (Cys^130^) and one tyrosine (Tyr^46^), with the latter likely interacting with Zn^2+^ through a water molecule [33]. Although the amino acid sequence identity of the Ts2631 endolysin and T7 lysozyme is relatively low at 26%, two histidines (His^30^, His^131^), a cysteine (Cys^139^) and a tyrosine (Tyr^58^) are conserved in the amino acid sequence of the Ts2631 endolysin (Fig 1). The aforementioned residues are also conserved in the sequence of a thermoresistant Ph2119 endolysin [12] and among all 12 amidase active PGRPs out of the 36 known peptidoglycan recognition proteins (PGRPs) [34].

**Fig 2:**
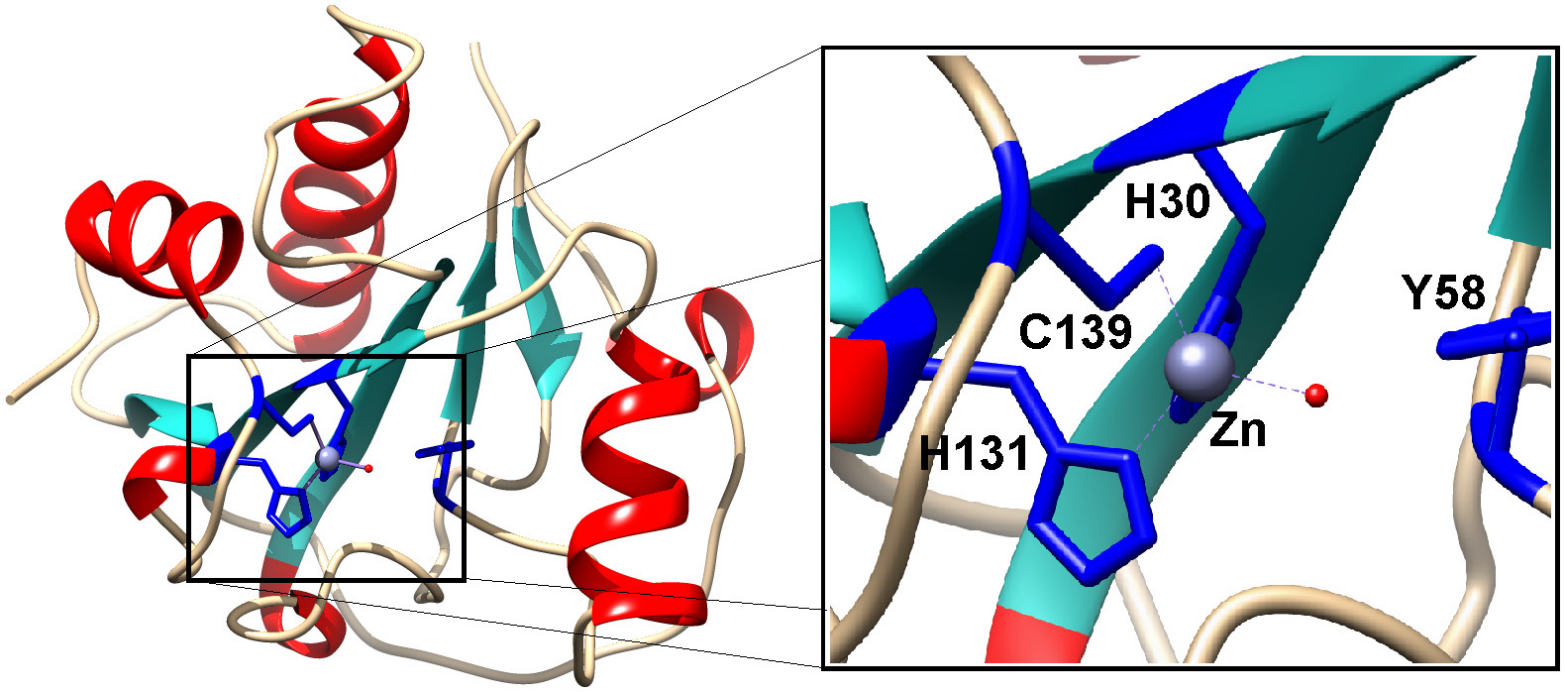
Structural model of Ts2631 endolysin. His30, Tyr58, His131, and Cys139, represented as blue sticks, are involved in Zn^2+^ binding. The secondary-structure elements alpha-helices, beta-strands, and loops are shown in red, cyan and grey respectively.

A molecular model of the Ts2631 endolysin based on *D*. *melanogaster* PGRP-LB structure (PDB: 1OHT) illustrates the three-dimensional arrangement of the potential Zn^2+^ binding site comprising His^30^, Tyr^58^, His^131^, and Cys^139^ (Fig 2, blue sticks). The structure of the Ts2631 endolysin is predicted to be very similar to its eukaryotic homologs with a central β-sheet and three peripheral α-helices (Fig 2) [15].

### Lytic activity of the Ts2631 endolysin

**Fig 3:**
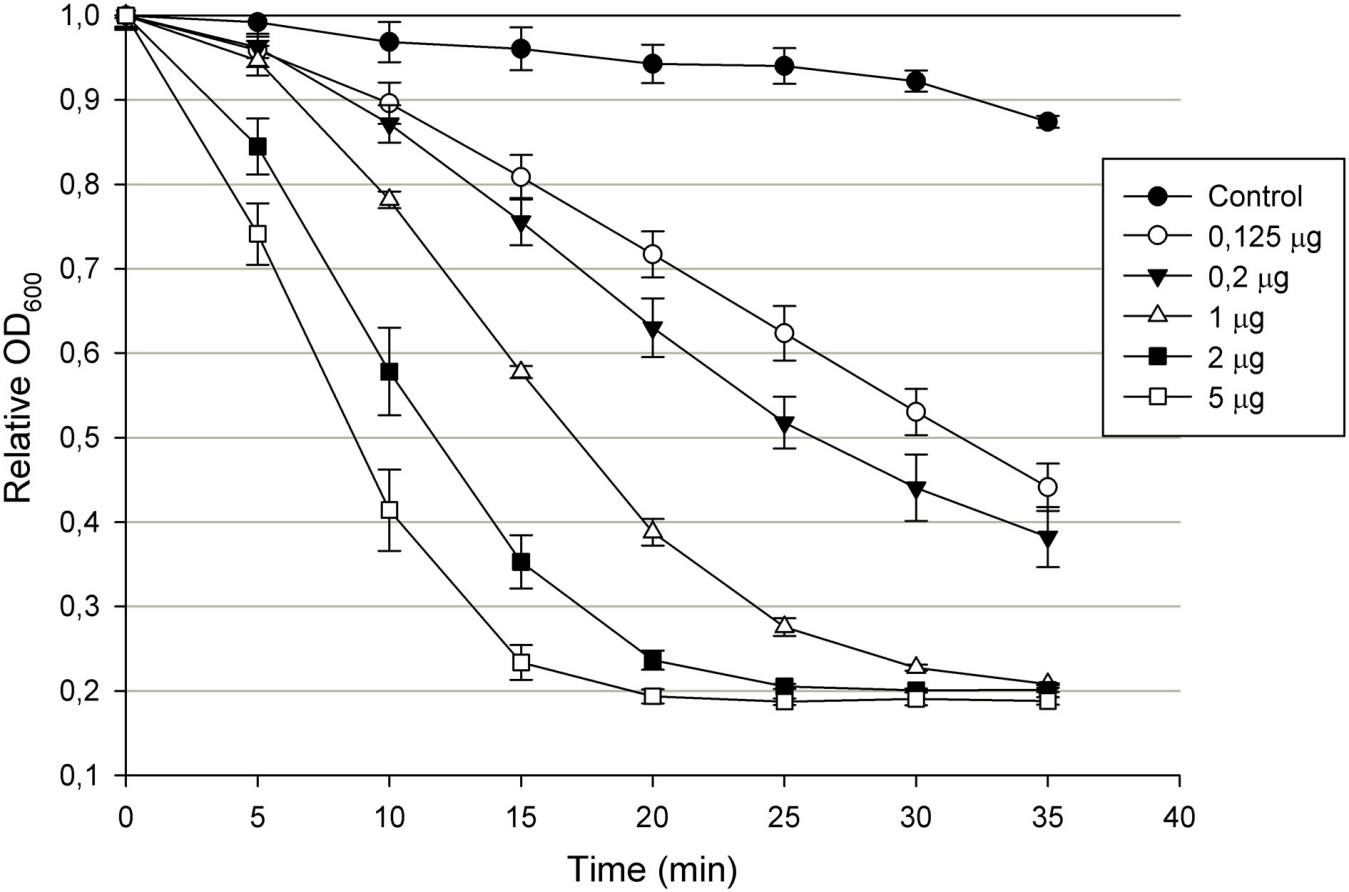
Lytic activity of Ts2631 endolysin. Different concentrations of Ts2631 endolysin were added to chloroform treated *T*. *thermophilus* HB8 DSM 579 cell suspension in 200 μl of 10 mM potassium phosphate buffer pH 8.0 and a decrease in OD_600_ was measured for 40 min in 5 min intervals. Results of the control (buffer only) were subtracted from the sample values. The experiment was repeated in triplicate; error bars indicate the standard deviation.

Recombinant Ts2631 endolysin with a N-terminal His-tag was purified from *E*. *coli* with use of metal ion affinity chromatography (IMAC) and showed strong lytic activity against *T*. *thermophilus* HB8 (S1A and S1B Fig). Fractionation of the Ts2631 endolysin by size-exclusion high-performance liquid chromatography revealed a single peak that eluted later than carbonic anhydrase (29 kDa), which indicated lack of aggregation, high purity of the protein and a monomeric nature (S1C Fig). The lytic activity of Ts2631 endolysin was also measured in a turbidity reduction assay against chloroform-treated cells of *T*. *thermophilus* HB8 used as a substrate. The lytic activity of the Ts2631 endolysin increased proportionally to the enzyme concentration until saturation was reached (5 μg of the protein after 20 min of the assay) (Fig 3).

While there is increased interest in the characterization of endolysins from phages infecting Gram-negative bacteria [35], there is still only a limited number of endolysins that, as an alternative to antibiotics, fight against Gram-negative bacteria [36]. The Gram-negative outer membrane (OM) is a barrier preventing entrance of endolysins into the bacterial cell and degradation of the peptidoglycan. To overcome this barrier, numerous chemical compounds that permeabilize the bacterial OM are used. The most commonly used are EDTA (removes OM stabilizing cations at a concentration range of 0.02–10 mM) and weak organic acids (2 mM citric acid and 5 mM malic acid) [36, 37]. Another method to permeabilize the Gram-negative OM is treating bacteria with chloroform-saturated Tris buffer. This method, which has been successfully used by others [29, 32], was used in the Ts2631 endolysin activity studies.

**Fig 4:**
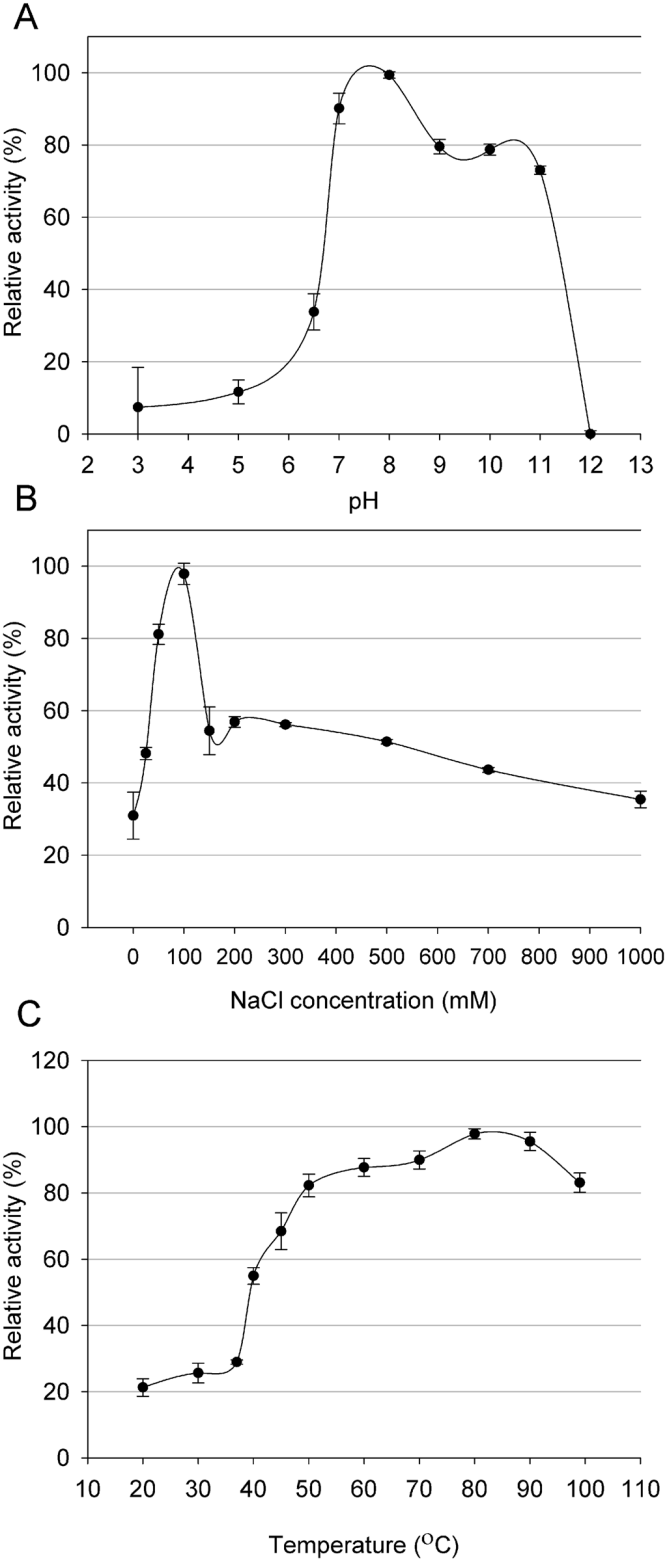
(A) Effect of pH, (B) NaCl and (C) temperature on the lytic activity of Ts2631 endolysin. Relative activity against *T*. *thermophilus* HB8 cells was calculated by comparing the lytic activity at specific condition with the maximal lytic activity among the dataset. Each experiment was repeated in triplicate; error bars indicate the standard deviation.

Thermophilic viruses represent an untapped source of enzymes with potential uses in biotechnology [12, 38]. In considering the putative antimicrobial potential of the Ts2631 endolysin, we carefully characterized its working optima. The endolysin activity was determined in a pH range of 3.0–12.0. The enzyme displayed activity exceeding 73% in a range from 7.0 to 11.00 with the optimum at pH 8.0. At more acidic conditions, the activity of the Ts2631 endolysin dropped significantly to the level of 11.7% at pH 5.0 (Fig 4A). The lytic activity of the Ts2631 endolysin was also measured at various NaCl concentrations up to 1 M in a 10 mM potassium phosphate buffer at pH 8.0. The endolysin exhibited optimum activity at 100 mM NaCl; however, the activity determined at a broad range of salt concentrations did not drop below 30%. The activity was 30.9% without NaCl and 35.5% with the highest salt concentration used (Fig 4B). The stability of the Ts2631 endolysin was also tested at different temperatures ranging from 20 to 99°C. The enzyme displayed optimum activity at the higher tested temperatures (over 82% of the enzyme activity was observed at temperatures from 50°C to 99°C) with the maximum at 80°C (Fig 4C). The biochemical properties of the Ts2631 endolysin (pH and optimal working temperature) are similar to another thermophilic endolysin from phage Ph2119, which infects *T*. *scotoductus* MAT2119 [12]. This is not surprising as the high amino acid sequence identity (74%) may reflect similar properties. However, the main difference is the salt requirement. Ph2119 endolysin preferred no NaCl for optimal activity while the Ts2631 endolysin had an optimal salt concentration of 100 mM. This finding makes the Ts2631 endolysin more attractive for applications in physiological conditions where the NaCl concentration is 130 mM [39].

Analysis of the molecular model of the Ts2631 endolysin suggested that the lytic activity of the protein, like in the cases of some other amidases, may depend on the presence of a metal ion in the enzyme catalytic site. To investigate the role of divalent metal cations on the lytic activity of the Ts2631 endolysin, the enzyme was treated with 5 mM EDTA for 30 min at 60°C to remove any residual metal cations. This treatment completely abolished the activity of the Ts2631 endolysin (Table 1). After overnight dialysis of the EDTA-treated sample against a reaction buffer (10 mM potassium phosphate buffer, pH 8.0), different metal cations (Co^2+^, Ni^2+^, Zn^2+^, Fe^2+^, Mn^2+^, Mg^2+^ and Ca^2+^) were added both to the protein and to the reaction buffer to achieve a final concentration of 0.1 or 1 mM. The negative control for each reaction contained reaction buffer supplemented with the specified metal ion. At the lower concentration (0.1 mM), Zn^2+^ was the most effective metal ion and reconstituted the Ts2631 endolysin activity to 80%. Mn^2+^ and Mg^2+^ showed no effect at 0.1 mM but higher concentrations of these cations (1 mM) restored Ts2631 lytic activity to 84.4% and 57.3%, respectively. Ca^2+^ was the most active metal ion at the 1 mM concentration resulting in activity that was 145% of the control. Zn^2+^ at the 1 mM concentration had no positive effect on the reaction. Therefore, it was concluded that the Ts2631 endolysin requires divalent metal ions, particularly Zn^2+^ (0.1 mM) or Ca^2+^ and Mn^2+^ (1 mM) for its lytic activity. Although, the restoration of Ts2631 endolysin activity above 100% in case of Ca^2+^ is surprising, similar results were reported previously for endolysins from *Listeria* bacteriophages, including HPL118, HPL500 and HPL35 [40]. This finding may be explained by increased interaction of the Ts2631 endolysin with a peptidoglycan backbone mediated by Ca^2+^ metal ions. In case of aminopeptidase A, a zinc metalloendopeptidase, a Ca^2+^ ion increases substrate affinity by binding to the acidic side chains of two aspartate residues of the enzyme and interaction with the negatively charged side chain of the substrate [41].

**Table 1:** Effect of divalent metal cations on lytic activity of Ts2631 endolysin. Activities are expressed as percentage in relation to the non-treated endolysin control. Values represent the mean ± standard deviation (n = 3).

	Ts2631 endolysin relative lytic activity (%)	
Control^a^	100.0 ± 3.75	
5 mM EDTA^b^	0	
Cation^c^:	0.1 mM	1 mM
Co^2+^	38.6 ± 8.8	29.0 ± 3.8
Ni^2+^	27.1 ± 6.8	17.7 ± 2.2
Zn^2+^	80.0 ± 4.0	0
Fe^2+^	32.3 ± 16.6	0
Mn^2+^	0	84.4 ± 17.5
Mg^2+^	0	57.3 ± 8.9
Ca^2+^	31.8 ± 10.6	145.1 ± 25.7

^a^ Lytic activities under standard conditions before EDTA treatment of Ts2631 endolysin.

^b^ Lytic activities of EDTA treated endolysins.

^c^ Lytic activities of EDTA treated and dialyzed endolysins against chloroform treated *T*. *thermophilus* HB8 cells supplemented with different divalent metal ions at 0.1 and 1 mM concentrations.

The substrate specificity of the Ts2631 endolysin was tested on various Gram-positive and Gram-negative bacteria having either Orn-, Lys- or DAP- type of peptidoglycan. The results obtained are shown in Table 2. Bacteria representing the Orn- type peptidoglycan, *T*. *scotoductus*, *T*. *flavus*, *T*. *thermophilus* HB8 and *D*. *radiodurans*, were effectively hydrolysed by the enzyme, and the lysis exceeded 79%. Additionally, the Ts2631 endolysin was active against mesophilic bacteria possessing DAP- type peptidoglycan: *E*. *coli*, *S*. *panama*, *P*. *fluorescens*, *S*. *marcescens* (lysis between 25.2 and 39.3%) and to a lesser extent against *B*. *cereus* and *B*. *subtilis* (lysis between 2.9 to 15.4%). The Ts2631 endolysin was not active against *C*. *sporogenes* and was barely active against Lys- type peptidoglycan bacteria with the hydrolysis reaching 3.0% only in the case of *M*. *luteus*. These results are in agreement with general observation that globular endolysins of phages infecting Gram-negative bacteria have a broader substrate spectrum [35]. The globular endolysins lack a CBD, which mediates specific binding to peptidoglycan and confers the endolysins high specificity sometimes resulting in restriction to specific bacterial host cells [42].

**Table 2:** Substrate specificity of Ts2631 endolysin. Relative activities are expressed as the percentage of activity to *T*. *thermophilus* HB8 DSM579. Values represent the mean ± standard deviation (n = 3).

Organisms:	Relative lytic activity (%)
*Thermus scotoductus* MAT2631	79.2 ± 2.7
*Thermus flavus* MAT1087	100 ± 3.3
*Thermus thermophilus* HB8 DSM 579	100 ± 5.8
*Enterococcus faecalis* KPD100-BA	0
*Escherichia coli* MG1655 ATCC 47076	26 ± 3.9
*Pseudomonas fluorescens* DSM 50090	46.2 ± 3.2
*Salmonella* serovar *Panama* KPD101-BA	39.3 ± 1.5
*Serratia marcescens* KPD102-BA	25.2 ± 3.9
*Bacillus cereus* ATCC 13061	15.4 ± 3.9
*Bacillus subtilis* ATCC 6633	2.9 ± 3.3
*Clostridium sporogenes* DSM 767	0
*Deinococcus radiodurans* ATCC 13939	96.7 ± 4.6
*Lactococcus lactis* KPD103-BA	0
*Micrococcus luteus* KPD104-BA	2.8 ± 3.0
*Staphylococcus aureus* ATCC 25923	0
*Staphylococcus epidermidis* ATCC 12228	0
*Staphylococcus intermedius* KPD105-BA	0

### Thermostability of Ts2631 endolysin

**Fig 5:**
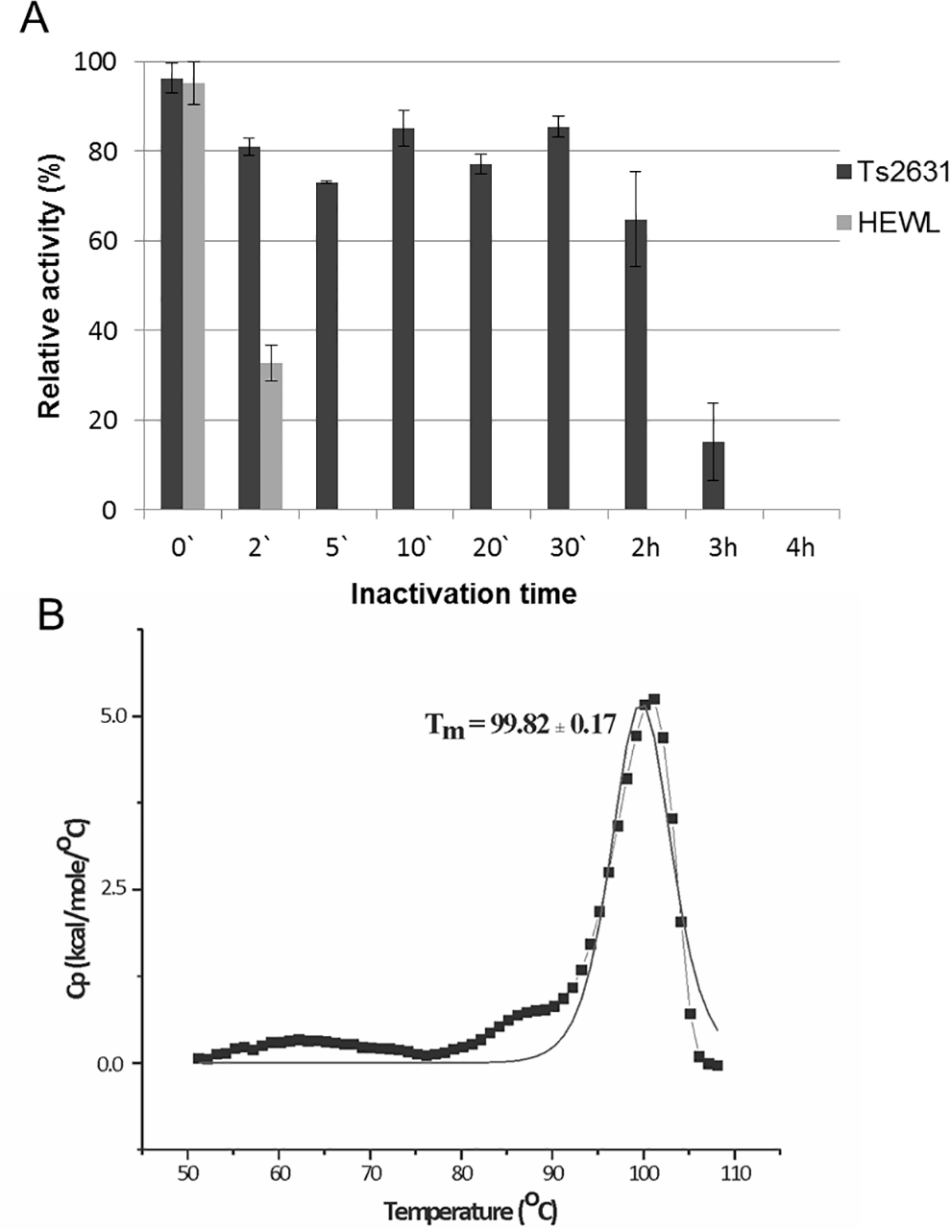
Examination of thermal stability of the Ts2631 endolysin. (A) Samples of Ts2631 endolysin (5 μg) and hen egg white lysozyme (HEWL, 5 μg) were incubated in 10 mM potassium phosphate buffer pH 8.0 at 95°C (0–4 h). Then samples were placed on ice before measuring their activity against *T*. *thermophilus* HB8 cells at optimal temperature (60°C for Ts2631 endolysin and 37°C for HEWL). The activity of Ts2631 endolysin and HEWL lysozyme is indicated as percentage relative to untreated samples. Each experiment was repeated in triplicate; error bars indicate the standard deviation. (B) Heat-capacity curves of Ts2631 endolysin determined by: DSC (black dotted line) and the fit to the two-state model [31] (black solid line) recorded in MES buffer, pH 6.0.

Thermoresistance of the Ts2631 endolysin was determined with the use of a standard turbidity assay of the protein samples (5 μg) heat treated at 95°C for the specified time periods. Each protein sample was subsequently cooled on ice and the residual activity was measured. HEWL was used as a control in this set of experiments. The Ts2631 endolysin retained 64.8% of its initial activity after 2 h at 95°C. However, when heated at 95°C for 3 h, the antimicrobial activity of Ts2631 dropped below 15.2% and was abolished after 4 h at 95°C (Fig 5A). At the same time, HEWL activity was completely abolished after 5 min at 95°C.

To assess the thermodynamic stability of the Ts2631 endolysin, differential scanning calorimetry analysis was performed. Analysis of the heat-capacity curve of the Ts2631 endolysin (Fig 5B) indicates that there is a heat-capacity peak at T_m_ = 99.82 ± 0.17°C. The peak on the heat capacity curve is sharp and has a height of 5.23 kcal/(mol × °C). The calorimetric heat of the first scan (ΔH_cal_) is 4.58 × 10^4^ cal mol^-1^. There are no significantly negative peaks in the heat-capacity curves, which demonstrates that the protein does not aggregate under the experimental conditions [43, 44]. However, the calorimetric measurements show that for the Ts2631 endolysin the process of thermal denaturation is irreversible. The calorimetric measurements until ~80°C show solely small, irregular changes with heat capacity (Cp) values of only approximately 0.4 kcal/(mol × °C) at ~63°C and about ~0.6 kcal/(mol × °C) at ~80°C. That suggests the lack of a folding—unfolding transition of the Ts2631 endolysin until ~80°C. Nevertheless, in the temperature range of 50°C–80°C the protein can undergo small interconverting changes of conformations, which are probably fluctuating monotonically with temperature. The melting temperature (T_m_) is the temperature at which 50% of the protein is unfolded and is the best parameter to describe proteins thermal stability [43,44]. T_m_ is not always available for hyperthermophilic proteins, which hampers correlation studies of the factors contributing to the protein stability and melting temperature [45]. The Ts2631 endolysin is the first endolysin from a thermophilic phage for which the T_m_ was determined. Nevertheless, the T_m_ is known for endolysins of mesophilic origin: *Salmonella* phage Lys68 endolysin (T_m_ of 44°C) [37], *Pseudomonas* phage ϕKMV lysin gp36C (T_m_ of 50.2°C) [46] and a few other endolysins that are not active at higher temperatures [47–49]. Both endolysins Lys68 and gp36C feature high thermoresistance, which is similar to the Ts2631 endolysin. The Lys68 endolysin maintains 54.7% of its residual activity at 80°C, whereas the gp36C retains over 50% of its activity after 1 h at 100°C [37, 29]. Still, the T_m_ values of both endolysins are significantly lower than the T_m_ of the Ts2631 endolysin (T_m_ of 99.8°C), indicating they have low thermodynamic stability. The difference between the aforementioned endolysins and Ts2631 enzyme lies in the reversibility of the thermal denaturation process. While Lys68 and gp36C endolysins refold to their native state after thermal stress [37, 46], the denaturation process of the Ts2631 endolysin is irreversible. It is not known if irreversible denaturation after prolonged heating is a general feature of endolysins of thermophilic origin, but investigation of the Ts2631 endolysin is the first step towards gaining better understanding of thermal stability of endolysins from *Thermus* phages. We strongly believe that studies of thermostable proteins, such as Ts2631, will not only lead to a greater theoretical understanding of the thermostability of the proteins but also to designing new antimicrobial cocktails including lytic enzymes with high kinetic and/or thermodynamic stability.

### Functional analysis of the catalytic residues

**Fig 6:**
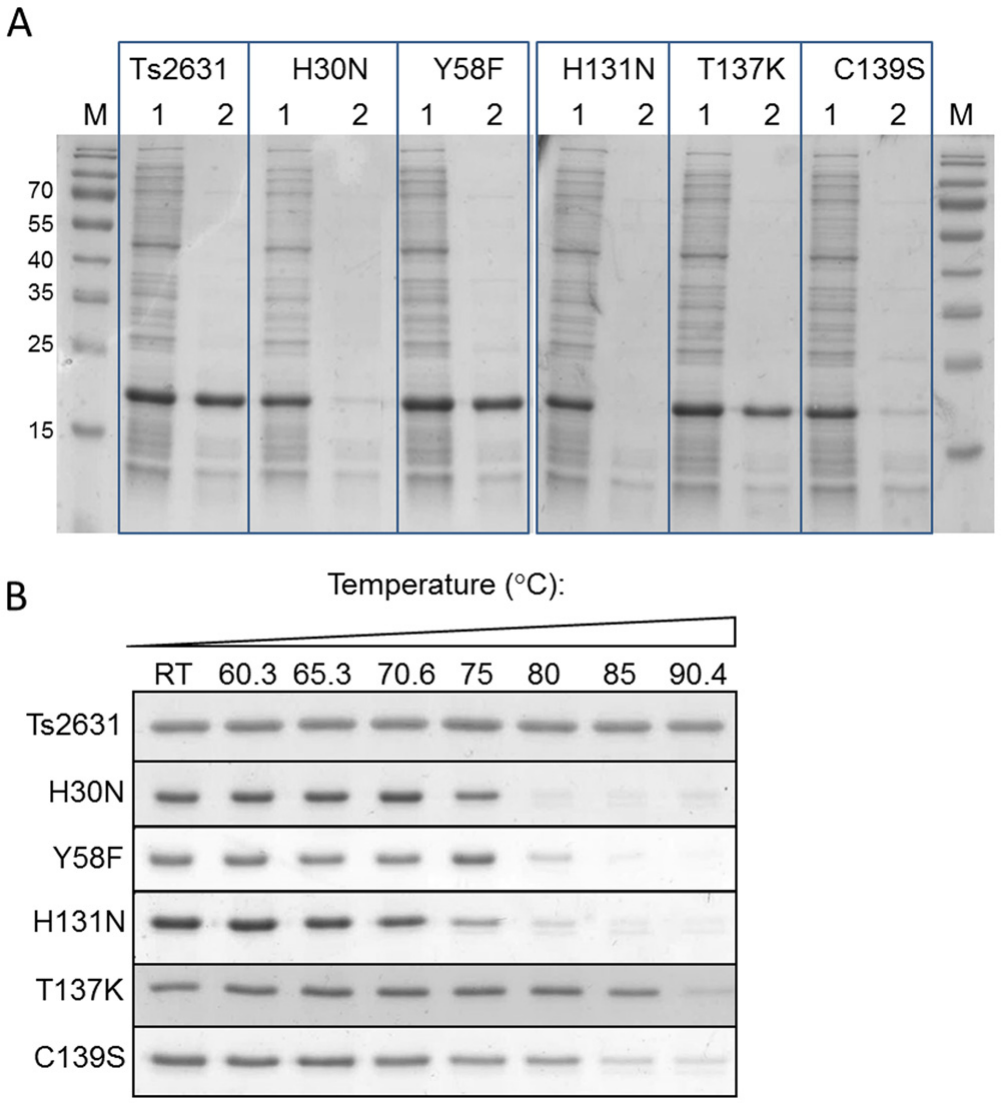
Thermostability of Ts2631 endolysin substitution variants. (A) Clear lysates of Ts2631 endolysin substitution variants (1) and supernatants after heat treatment (2) were mixed with Laemmli buffer and loaded on 12.5% SDS-PAGE. Ts2631 endolysin was used as an experiment positive control. Gels were stained with Coomassie Blue R-250 and visualized with use of Gel Doc XR+ Imager (BioRad). (B) Ts2631 endolysin and its substitution variants (20 μl) at concentration 0.5 mg ml^-1^ were incubated in thermocycler at indicated temperature gradient for 15 min. Samples were subsequently centrifuged (10,000 × g; 20 min; 4°C) to remove aggregated proteins. Supernatant (6 μl) from each fraction was mixed with Laemmli buffer and loaded on 12.5% SDS-PAGE. Densitometric analysis was performed with use of Image Lab 5.1 BETA software (Bio-Rad). At 75°C 73% of H131N and more than 32% of H30N and C139S aggregate. At 85°C native enzyme and T137K variant were soluble, while at 90.4°C only Ts2631 endolysin remained in the supernatant.

*In silico* analysis of both the predicted Ts2631 amino acid sequence and its tertiary structure revealed the existence of several conserved residues that may play a role in the endolysin activity (Figs 1 and 2). The presence of a cysteine residue in a Zn^2+^ binding tetrad is characteristic for eukaryotic PGRPs having amidase activity. In non-amidase PGRPs that do not bind Zn^2+^, the cysteine is substituted with a serine residue [13]. In the alignment, the non-amidase active PGRPs are represented by PGRP-SA and PGRP-HOLDI, which have serine residue in place of the conserved cysteine (Fig 1). PGRP-SA and PGRP-HOLDI only recognize but do not cut bacterial peptidoglycan. In the present study, we investigated the influence of the C139 residue on Ts2631 endolysin lytic activity by analysis of a C139S protein variant. In addition, it should be noted that all non-phage encoded PGRPs have a conserved Thr residue (Thr137 in case of Ts2631 endolysin) that corresponds to the lysine residue in the sequences of mesophilic phage lytic enzymes (Fig 1). It was previously reported that in the case of the T7 lysozyme and *D*. *melanogaster* PGRP-LB that the reverse substitutions (K128T and T158K, respectively) lead to a considerable drop of activity of both lytic enzymes [24,33]. Because the Ts2631 endolysin is a rare example of a phage lytic enzyme with a threonine residue in the potential active site, we investigated the activity of a T137K variant. The T7 lysozyme, one of the best studied examples of a mesophilic phage lytic enzyme, is a bi-functional protein that combines amidase activity with T7 RNA polymerase inhibition activity. In T7 lysozyme activity studies, the residue substitutions Y46F, K128T and H36N did not or only slightly decreased the inhibitory activity of the enzyme. The obtained results suggested that the introduced changes (substitutions) did not significantly affect the protein structure [33]. Given the above reasons, and bearing in mind that in the case of the Ts2631 endolysin that the His^30^, Tyr^58^, Cys^139^ and His^131^ residues may form the active centre of the protein (Fig 2), we decided to substitute those residues and perform activity tests of the purified Ts2631 endolysin variants. The chosen variants were as follows: His30Asn (H30N), Tyr58Phe (Y58F), His131Asn (H131N), Thr137Lys (T137K) and Cys139Ser (C139S). The standard Ts2631 endolysin purification protocol was used and includes a cell lysate heat treatment step (15 min 75°C) at which most *E*. *coli* thermolabile proteins are denatured and removed by subsequent centrifugation. Heat treatment of the clear lysate of the Ts2631 endolysin substitution variants resulted in partial aggregation of the H30N, H131N and C139S variants (Fig 6A). These preliminary data prompted us to determine the temperature at which the Ts2631 substitution variants are no longer soluble. After a 15 min heat treatment at 70.6°C, all of the substitution variants remained in supernatant, whereas at 75°C, as determined by densitometric analysis, 73% of the H131N variant and more than 32% of H30N and C139S variants aggregate (Fig 6B). At 85°C, only the native enzyme and the T137K variant were soluble, while at 90.4°C only the native Ts2631 endolysin remained in the supernatant (Fig 6B). Therefore, we decided to omit the heat pre-treatment step during protein purification using metal-affinity chromatography. The native Ts2631 endolysin (used as a control in this set of experiments) and the substitution variants were purified to near homogeneity as estimated following SDS-PAGE analysis (data not shown). The activity tests with the *T*. *thermophilus* HB8 substrate show that all of the substitutions greatly reduced endolysin activity. Compared to the 100% activity of native Ts2631 endolysin, after 15 min no activity of the substitution variants was observed (data not shown). After prolonged incubation (1 h) with permeabilized *T*. *thermophilus* cells, the activity of the Ts2631 endolysin variants did not exceed 18%. Specifically, the activity of the Ts2631 endolysin variants was 17.2% for H30N, 10.3% for Y58F, 3.0% for H131N, 11.8% for T137K and 11.8% for C139S (Table 3).

**Table 3:** Lytic activity of Ts2631 endolysin variants. Substitutions were generated by site-directed mutagenesis of a clone of Ts2631 endolysin in the pET15b vector (Novagen) using oligonucleotides with a degenerate sequence at the target codon. Mutations are identified by the one-letter code for the wild-type amino acid followed by the residue number and then the one-letter code for the mutant amino acid. Amidase activity was measured in a standard turbidity assay against chloroform-treated substrate *T*. *thermophilus* HB8. Activities are expressed as percentage in relation to the native Ts2631 endolysin control. Values represent the mean ± standard deviation (n = 3).

Variants:	Relative lytic activity (%)
Ts2631	100 ± 1.09
H30N	17.17 ± 1.0
Y58F	10.30 ± 2.7
H131N	2.99 ± 5.8
T137K	11.76 ± 2.4
C139S	11.83 ± 4.3

Zinc-binding sites in proteins often adopt tetrahedral coordination geometry formed by the sulphur of cysteine, the nitrogen of histidine or the oxygen of aspartate and/or glutamate [50]. In the case of the T7 lysozyme, the role of the glutamate side chain is likely fulfilled by a tyrosine residue through a water molecule [33]. This assumption is also likely true for PGRP-LB [24]. A water molecule is a critical component of the catalytically active zinc sites. On the other side of the site, direct coordination to four residues is typical in proteins where a Zn^2+^ ion plays a structural role [51]. In thermolysin from *Bacillus thermoproteolyticus*, a metalloprotease, a Zn^2+^ ion plays a catalytic but not a structural role, and it does not contribute to the unusually high thermal stability of the enzyme [52]. However, a structural and functional role for Zn^2+^ is known for other thermostable enzymes, namely bioremediase [53]. All Ts2631 endolysin substitution variants were soluble at 60°C, which is the temperature that the lytic activity tests were performed (Fig 6B). Therefore, we postulate the studied residues have a direct role in catalytic activity of the enzyme. Surprisingly, the H30N, H131N and C139S variants were unstable at temperatures ≥75°C, which indicates that those residues play an additional role in the stabilization of the Ts2631 endolysin structure at elevated temperatures. Preliminary DSC experiments of EDTA-treated Ts2631 endolysin also showed that the T_m_ of EDTA-treated protein is 81.34 ± 0.1°C which is lower than the corresponding T_m_ of the native protein (99.82 ± 0.17°C), suggesting a role of Zn^2+^ in protein thermal stability (data not shown).

## Supporting Information

S1 Fig Purification and activity of Ts2631 endolysin. (TIF)

S1 Table PCR primers used in this study. (DOCX)
