# Supplementary figures and images for: Biochemical Characterization and Validation of a Catalytic Site of a Highly Thermostable Ts2631 Endolysin from the Thermus scotoductus Phage vB_Tsc2631
Source: PLoS One. 2015 Sep 16;10(9):e0137374. doi: 10.1371/journal.pone.0137374 (PMC4573324; doi:10.1371/journal.pone.0137374)

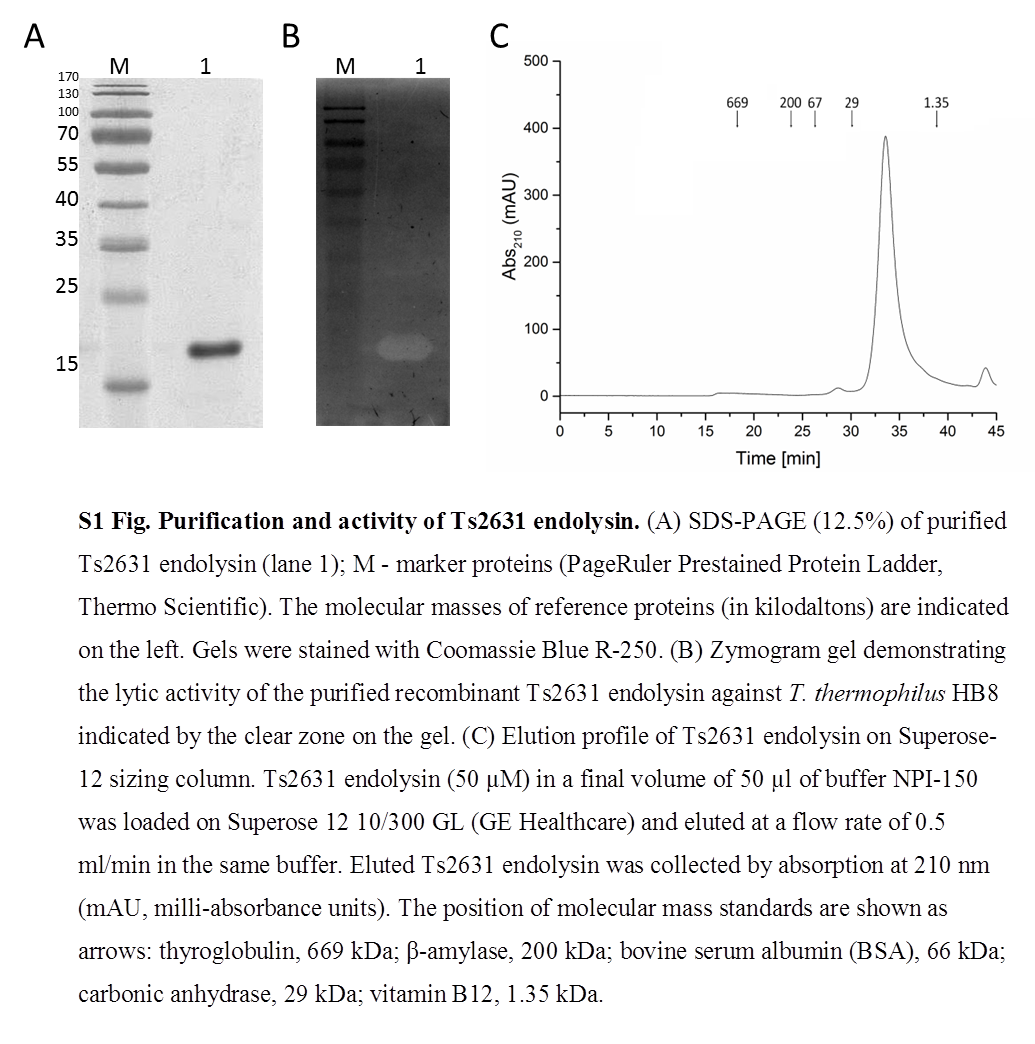

Supplement: S1 Fig — (TIF) [file pone.0137374.s001.tif]
