# Supplementary material for: Biochemical Characterization and Validation of a Catalytic Site of a Highly Thermostable Ts2631 Endolysin from the Thermus scotoductus Phage vB_Tsc2631
Source: PLoS One. 2015 Sep 16;10(9):e0137374. doi: 10.1371/journal.pone.0137374 (PMC4573324; doi:10.1371/journal.pone.0137374)
Supplement: S1 Table — (DOCX) [file pone.0137374.s002.docx]

**S1 Table. PCR primers used in this study**

| Primer name: | | | Oligonucleotide sequences (5 ́→ 3 ́): | Annealing temperature: | |  |
| --- | --- | --- | --- | --- | --- | --- |
| **Cloning of *ts2631* gene** | | | | | |  |
| Ts2631-nde-f | | CGGGGAATTCCATATGAGGATACTAGAACCATGGAA | | | 64.4 °C |  |
| Ts2631-bam-h-r | | CGCGGATCCTTACTGACCACCTCCCCCCT | | | 68.6 °C |  |
| **Site-directed mutagenesis of *ts2631* gene** | | | | | Codon change: |  |
| H30N_F | CATTACGTTGTCCTGAATCACACGGCGGGGCCG | | | | CAC → AAT |  |
| H30N_R | CGGCCCCGCCGTGTGATTCAGGACAACGTAATG | | | | CAC → AAT |  |
| Y58F_F | GGTGGCCCCATATCGGTTTTCACTATCTGGTCTACCG | | | | TAC → TTT |  |
| Y58F_R | CGGTAGACCAGATAGTGAAAACCGATATGGGGCCACC | | | | TAC → TTT |  |
| H131N_F | GTGGGTACAAGGTTTTTGTTCAGTACAAAAAGCGCCTTA | | | | CAC → AAC |  |
| H131N_R | TAAGGCGCTTTTTGTACTGAACAAAAACCTTGTACCCAC | | | | CAC → AAC |  |
| T137K_F | GCCCAGGGCATTCTTTGGGTACAAGGTTTTTGTG | | | | ACA → AAA |  |
| T137K_R | CACAAAAACCTTGTACCCAAAGAATGCCCTGGGC | | | | ACA → AAA |  |
| C139S_F | CGCCCAGGGCTTTCTGTGGGTACAAGG | | | | TGC → AGC |  |
| C139S_R | CCTTGTACCCACAGAAAGCCCTGGGCG | | | | TGC → AGC |  |

Primers for cloning of *ts2631* gene were design with use of Primer3plus software. Primers for site-directed mutagenesis of *ts2631* gene were design with use of QuikChange Primer Design Program available online at http://www.stratagene.com/qcprimerdesign. Underlined sequences indicate cleavage sites for NdeI and BamHI, respectively.
